# Vitamin D Deficiency and Gingival Enlargement: A Case Report

**DOI:** 10.7759/cureus.37378

**Published:** 2023-04-10

**Authors:** Akash Meher, Manish Goel, Ritika Jain, Neha Dhadse, Kapil Paiwal

**Affiliations:** 1 Department of Pedodontics and Preventive Dentistry, Daswani Dental College and Research Center, Kota, IND; 2 Department of Periodontology and Implantology, Rajasthan Dental College and Research Center, Jaipur, IND; 3 Department of Oral and Maxillofacial Pathology, Daswani Dental College and Research Center, Kota, IND

**Keywords:** vitamin d supplementation, innate immune system, pediatric case report, gingival overgrowth, vitamin-d deficiency

## Abstract

The occurrence of vitamin D insufficiency is rising constantly, and most pediatric patients are below the required levels. Individuals with vitamin D deficiency are more susceptible to inflammatory diseases because it reduces their immunity. The role of vitamin D deficiency in gingival enlargement has been reported in the literature. In this case report, we are describing a case in which a vitamin D supplement has resolved the gingival enlargement significantly without any invasive procedure.

A 12-year-old boy reported a chief complaint of swollen gums in the upper and lower front teeth region. On clinical examination, there was minor surface plaque and calculus along with the formation of pseudopockets, but there was no clinical attachment loss. The patient has been advised to undergo laboratory tests for a complete blood profile, including a vitamin assessment. The patient reported after two and a half months with a gingivectomy on the first quadrant at a private clinic. They reported back to us because they didn’t want the same trauma from surgery again and wanted a more conservative treatment option. So, on the basis of the reassessment of reports, vitamin D deficiency was confirmed, and treatment was started with 60,000 thousand I/U of vitamin D supplement weekly and advised for sunlight exposure with minimal clothing. There was a significant decrease in enlargement observed after the six-month follow-up period. Vitamin D supplements can be a more conservative treatment option for gingival enlargement of unknown etiology.

## Introduction

“Gingival overgrowth” or “gingival enlargement” is one of the different types of periodontal diseases. Gingival enlargement, which is a heterogeneous disease, can be classified as (1) inflammatory enlargement, (2) drug-induced enlargement, (3) overgrowth associated with systemic diseases, (4) neoplastic overgrowth, and (5) false enlargement. Now it has been proven that there is a relevant relationship between nutritional deficiency and oral disease, especially gingivitis [1].

Vitamin D is a group of fat-soluble vitamins that was first discovered in 1914 by McCollum and his co-workers as an antirachitic agent [2]. The most efficient way to obtain vitamin D is through natural sunlight. Mid-day exposure of the arms and legs of fair-skinned Caucasians for 5-10 minutes can produce up to 3000 IU of vitamin D3 in the epidermis of the skin. In the case of whole-body exposure, which can increase up to 10,000 IU [3]. But nowadays, factors like limited outdoor activities, sunscreen application, pollution, and clothing have caused a reduction in ultraviolet B radiation (UVB) exposure, creating a need for vitamin supplementation. Patients with gingivitis can be supplemented with a weekly single dose of 50,000 IU of vitamin D for two to three months to achieve relevant effects [4].

Vitamin D plays an important role in maintaining oral immunity and the integrity of the periodontium by utilizing autocrine/intracrine pathways associated with gene signaling expression, protein synthesis, hormone synthesis, regulation of immune-inflammatory responses, and self-turnover [5].

The perio-protective role of vitamin D may be due to the ability of vitamin D to stimulate the innate immune response through the production of antimicrobial peptides, such as cathelicidin and β-defensin, which will strengthen epithelium and which makes it difficult for pathogens to breach it. Vitamin D also has the ability to shape the adaptive immune response by selectively stimulating specific T-helper cell subsets, which may help in the resolution of inflammation [6].

In this case report, vitamin D supplementation of 60,000 IU weekly and adequate sun exposure have resolved the gingival enlargement significantly without any invasive procedure.

## Case presentation

A 12-year-old boy reported to the Department of Pediatric and Preventive Dentistry at the Daswani Dental College and Research Center in Kota with a chief complaint of swollen gums in the upper and lower front tooth regions and gave a history of gradually increasing gum size to its present status in the last one year.

**Figure 1:**
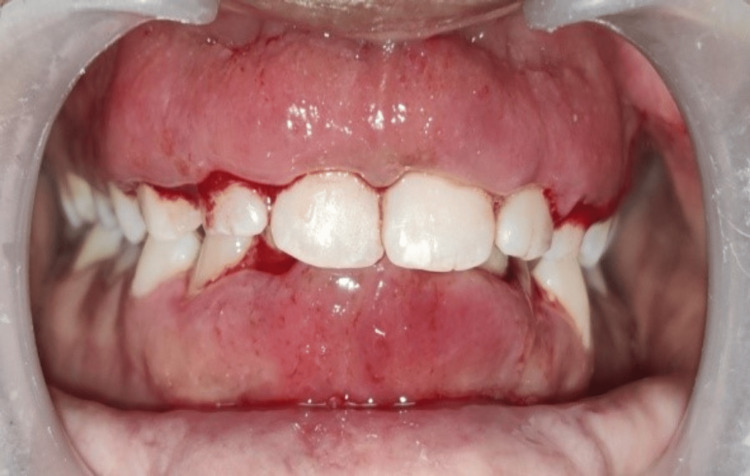
Preoperative clinical picture of the patient.

On the clinical examination, there was plaque accumulation with a score of 2 on the Silness-Loe plaque index with the formation of pseudopockets, and a probing pocket depth of 5 mm. The pubertal gingivitis was ruled out because of other clinical findings and a loss of clinical attachment. Minor crowding in mandibular anterior teeth with class 1 fremitus was also observed (Figure 1).

**Figure 2:**
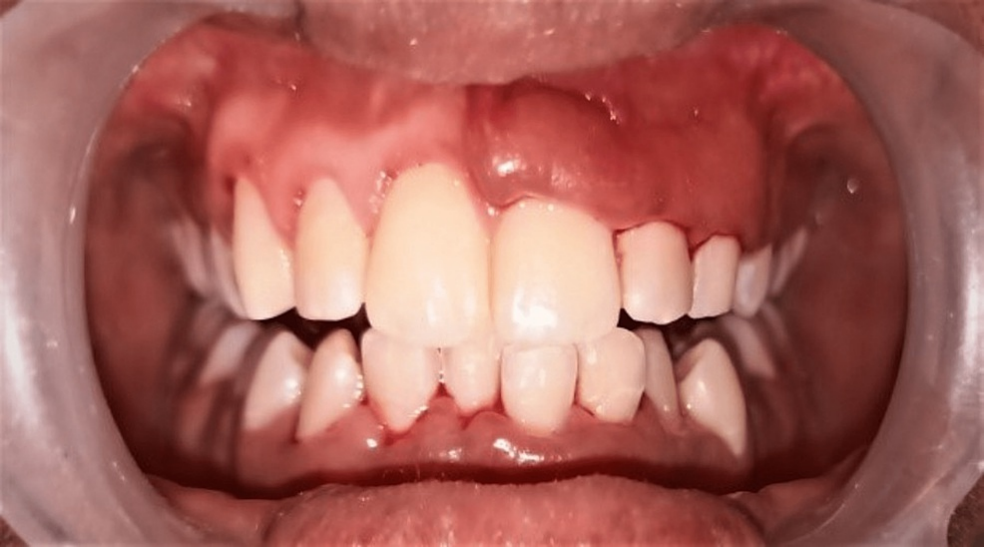
Patient reported after two and a half months with gingivectomy on the first quadrant.

The patient reported to the department after two and a half months after the gingivectomy was done on the first quadrant by a private practitioner and reported back to the department as they didn’t want the same trauma from surgery again and requested a conservative treatment option (Figure 2).

The differential diagnosis of this condition is pubertal gingivitis, vitamin C deficiency gingival enlargement, inflammatory gingival enlargement, and false gingival enlargement. But according to blood reports and vitamin reports, everything was within the normal range except for the deficiency of vitamin D, which was 12 ng/ml, i.e., a deficient level.

**Figure 3:**
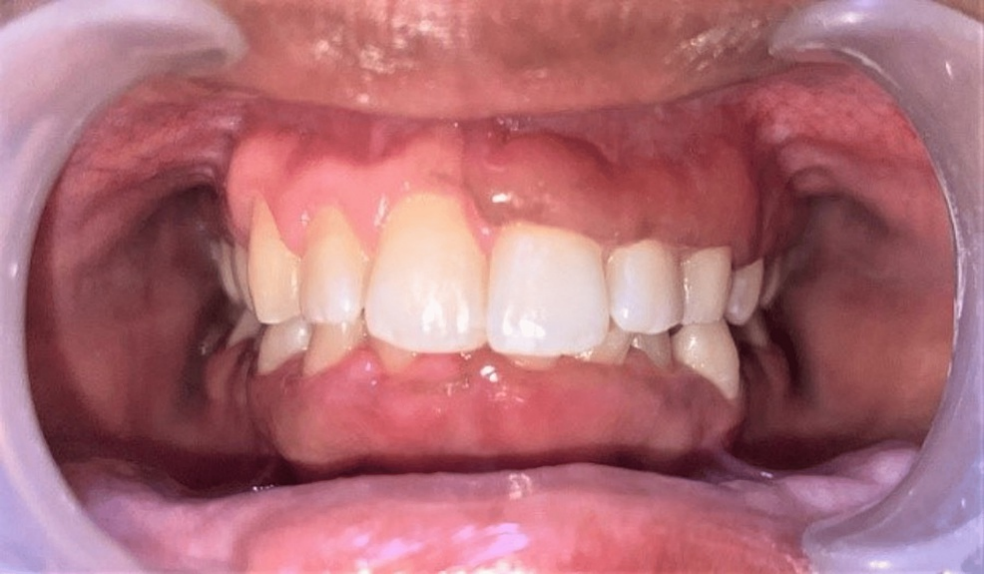
Two months of follow-up.

**Figure 4:**
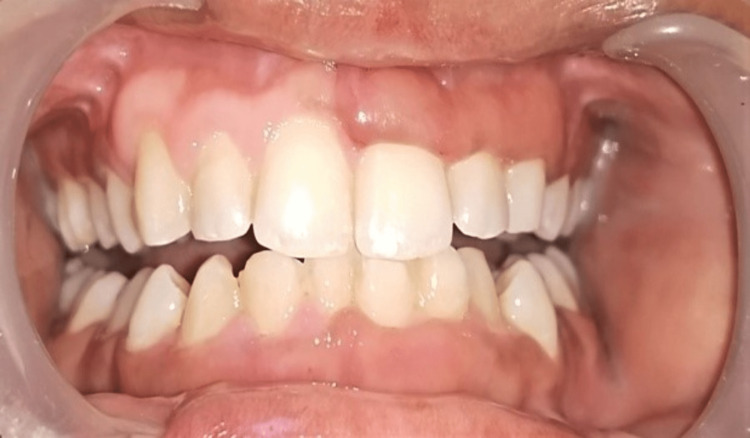
Four months of follow-up.

**Figure 5:**
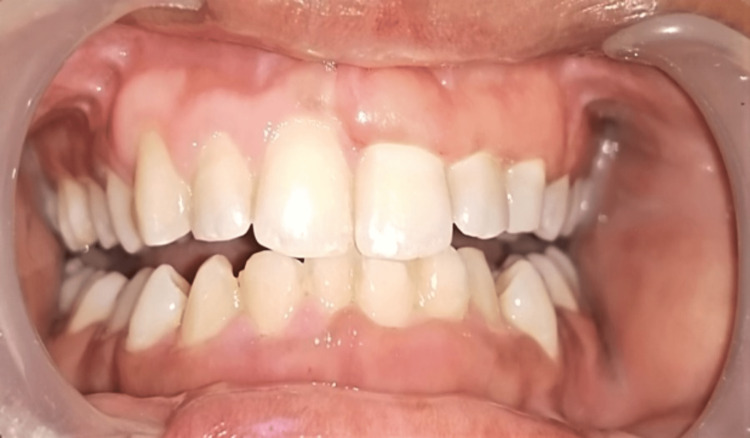
Six-month follow-up.

Literature has suggested an association between vitamin D and gingival enlargement by reducing the accumulation of plaque on the tooth surface and reducing the inflammation of gingival tissue. So our primary diagnosis was vitamin D deficiency, and we opted for vitamin D supplementation as treatment of this condition with every two-month follow-up (Figures 3, 4, 5).

Case management

Vitamin D supplementation of 60,000 IU weekly with whole-fat milk was prescribed, and the patient was advised to sun exposure with minimal clothing in the morning for at least half an hour.

Clinical outcomes

After every two months of follow-up, a reduction in gingival enlargement was observed, and after six months, significant changes were seen, i.e., decreased in the size of the enlargement, and in the blood report, there was an increase in the serum vitamin D level, i.e., 105 ng/ml.

## Discussion

Our current lifestyle, due to decreasing sun exposure and decreasing proper nutritional food intake, has ultimately led to a deficiency of vitamin D. Vitamin D is absorbed by the body in two forms: D2 (ergocalciferol) and D3 (cholecalciferol). The majority of vitamin D is synthesized by skin exposure to UVB light through the photoconversion of 7-dehydrocholesterol to vitamin D3. The rest of the vitamin D is absorbed by the body from plant sources in the form of D2 and from animal sources in the form of D3. Both D2 and D3 are biologically inactive and are converted into 25(OH)D by the liver, which is then converted to 1,25(OH)2D mainly by proximal tubular cells, renal nephrons, and some other organs like the prostate gland, breast, and colon, etc., which is the biologically active form of vitamin D. This active form of vitamin D widespread and exerts most of its anti-inflammatory, antibacterial, and antiviral activities through a transcription factor named vitamin D nuclear receptor (VDR) by forming 1,25(OH)2D/VDR [7].

Literature has reported the relationship of vitamin D deficiency with various oral conditions and its relation with various conditions like gingival diseases, periodontal diseases, bone loss, and many other infectious and chronic inflammatory diseases [1,4,5,6].

The statistical correlation between vitamin D and periodontal disease as observed in cross-sectional studies can be explained by several biological mechanisms. The biologically active form of vitamin D, i.e., 1,25 (OH)2D regulates calcium and bone homeostasis and can reduce the resorption of alveolar bone by increasing its mineral density. 1,25 (OH)2D/VDR signaling pathway reduces the bacterial burden by inducing the production of cathelicidin and β-defensin by activated keratinocytes, monocytes, and macrophages of the periodontium. 1,25 (OH)2D/VDR signaling may reduce the inflammatory process of periodontal diseases induced by bacteria by suppressing cyclooxygenase-2 (COX-2) and prostaglandin pathways. This signaling pathway also mediates the proliferation and differentiation of keratinocytes and also recruits macrophages during the inflammatory phase of tissue repair, which in turn helps wound healing [8].

Wang et al. showed that vitamin D may decrease the number of live *Porphyromonas gingivalis* through active autophagocytosis and might alleviate the inflammatory burden of periodontitis in rodent models, apparently through systemic T-helper cells [9].

The Institute of Medicine (IOM) (USA) in 2010 recommended a dietary intake of vitamin D intake of 600 IU/day for the age of one to thirteen years of children [10]. But according to some experts, this recommendation may prevent clinical vitamin D deficiency (less than 20 ng/mL) and insufficiency (less than 30 ng/mL), but these levels are far lower than what is necessary to attain adequate vitamin D status controlled by autocrine processes [11]. According to the Endocrine Society, the upper limit for vitamin D is 10000 IU [12], and the IOM (USA) has suggested that 10,000 IU/day of vitamin D supplementation has no adverse effect on the body [13].

According to the study by the third National Health and Nutrition Examination Survey (NHANES III-USA), it was observed that individuals with the highest levels of vitamin D experienced 20% less bleeding on probing than those with the lowest levels [14]. Hiremath et al. concluded in their study that there is a dose-dependent anti-inflammatory effect of vitamin D on gingivitis. They also concluded that a higher dose of vitamin D gives early results [15]. Nesterova et al. advocated pharmaceutical intervention to correct hypovitaminosis D as part of the treatment of periodontal diseases [16].

In this case report, the intraoral diagnosis of gingival enlargement without any identifiable cause and the laboratory diagnosis of vitamin D deficiency made it possible to treat this condition with a vitamin D supplement. The local factors, i.e., minor tooth crowding and poor oral hygiene, that may mediate inflammatory gingival enlargement do not fully explain the massive gingival enlargement in our patient. In the presence of oral biofilm, which exacerbates gingival inflammation and thus promotes gingival enlargement, a vitamin D deficit may operate as a modifying factor. Supplemental vitamin D has been found in numerous studies to lessen the recurrence of gingival hyperplasia. In this case, also improved baseline levels of serum vitamin D after every three-month follow-up also improved pocket depth from 5 mm to 3 mm. It has shown constantly improved gingival condition, which supports the above-stated mechanism of action of vitamin D on gingival health by decreasing the accumulation of plaque on the tooth surface, so it reduces the gingival inflammation and its perioprotective role by preventing bacterial percolation into the periodontal tissue and also positively correlated the higher dose supplementation, which improves the gingival condition.

## Conclusions

In this case, vitamin D deficiency has been attributed as one of the etiological factors for gingival enlargement. The response of the vitamin D supplement has proven to have a positive correlation with decreased probing pocket depth from 5 mm to 3 mm, inflammation, and gingival enlargement. Hence, in cases of gingival enlargement where the major local factors are absent, the treatment option of vitamin D supplementation can be considered. The limitation of the study includes treating the gingival enlargement with vitamin D supplementation in a single case only, so further studies with a larger sample size are needed.
